# Serum Level of CC-Chemokine Ligand 18 Is Increased in Patients with Non-Small-Cell Lung Cancer and Correlates with Survival Time in Adenocarcinomas

**DOI:** 10.1371/journal.pone.0041746

**Published:** 2012-07-25

**Authors:** Till Plönes, Alexander Krohn, Meike Burger, Hendrik Veelken, Bernward Passlick, Joachim Müller-Quernheim, Gernot Zissel

**Affiliations:** 1 Department of Thoracic Surgery, University Medical Center Freiburg, Freiburg, Germany; 2 Department of Oncology and Haematology, University Medical Center Freiburg, Freiburg, Germany; 3 Department of Pneumology, University Medical Center Freiburg, Freiburg, Germany; 4 Department of Haematology, Leiden University Medical Center, Leiden, The Netherlands; University of Colorado School of Medicine, United States of America

## Abstract

CC-chemokine ligand 18 (CCL18) is mainly expressed by alternatively activated macrophages and DCs and plays an important role in lung fibrosis, arthritis and other diseases. Here CCL18 was measured in sera of 31 healthy volunteers and 170 patients with lung cancer and correlated these data with histology, tumor stage and clinical parameters. Mean CCL18 serum level of the patients with non-small-cell lung cancer was 150(857) ng/ml vs. 32(61) ng/ml in the healthy control group. Patient groups differ significantly according their histology (adenocarcinoma 143(528) ng/ml vs squamous cell carcinoma 187(857) ng/ml, p<0.02). In addition, we found a significant difference between patients with lower versus higher T-stage (p<0.003). Receiver operating characteristic (ROC) analyses revealed a cutoff point of 83 ng/ml (area under the curve (AUC): 0.968; p<0.0001) to discriminate between healthy controls and non-small-cell lung cancer patients. ROC analyses to discriminate between patients, who died because of cancer related death and those who died for other reasons did not lead to a valid AUC. To stratify the tumor patients, a criterion value plot was performed leading to a point of equal sensitivity and specificity (54%) of 162 ng/ml. Patients with a CCL18 serum level higher than 160 ng/ml had a mean survival time of 623 days. In contrast, those in patients with a baseline level between 83 ng/ml and 160 ng/ml the mean survival time was 984 days (p<0.005). Survival-analysis revealed in adenocarcinoma a mean survival of 1152 days in the group below 83 ng/ml. In the median group the mean survival time was 788 days and in the group with the highest levels the mean survival time was 388 days (p<0.001). In contrast, we found no correlation between the FEV1 and the CCL18 baseline level. In conclusion, in patients suffering from adenocarcinoma increased serum CCL18 levels predict a diminished survival time.

## Introduction

Lung cancer is the leading cause of cancer-related mortality and is one of the most important malignant neoplasms because of its high prevalence and increasing incidence [1]. Nearly 80% of all lung cancers are histological defined as non-small-cell cell lung cancer (NSCLC). Despite of advantages in new technologies and developing of new drugs contributing to a much earlier diagnosis and a more sufficient treatment, NSCLC remains a life threatening disease. The overall 5-year survival for NSCLC patients is still low and even in the early stages of the disease the recurrence rate is relatively high [2], [3]. The poor prognosis is due to the highly aggressive behavior expressed by fast progressive tumor growth and early metastasis. Although the exact mechanism of carcinogenesis and metastasis in NSCLC are still unknown, the microenvironment of the tumor seems to play a key role in the development of this malignant disease and the dissemination of the tumor cells. The microenvironment of solid tumors is a complex mixture of cellular and non cellular factors [4]. Especially the immune cells located in the surrounding of the tumor and the chemokine-crosstalk promote the growth of tumor cells and their spreading [5]. Tumor infiltrating macrophages which are also known as tumor associated macrophages (TAM) are one of the most important subgroup of immune cells in the tumor microenvironment and represent up to 50% of the tumor mass. Some studies demonstrate a significant correlation between the positive number of TAMs and a worse prognosis in malignant diseases [6]. Initially monocytes are recruited from the circulating blood to the tissue and differentiate in the two phenotypes M1- or M2 macrophages. The M1 phenotype is associated with pro-inflammatory pathways characterized by the release of pro-inflammatory cytokines and increased microbial killing. The M2 phenotype shows a proangiogenic, prometastatic and protumoral activity and is associated with tissue remodeling. TAMs are mostly M2-Macrophages which secrete a specific pattern of chemokines [6]–[8]. They are characterized by release of IL-10, CCL17, CCL22, IL-1Ra, and CCL18 [9]. The CC-Chemokine CCL18, previously named “pulmonary and activation-regulated chemokine” is strongly expressed in human lungs and less in other lymphatic tissue like lymph nodes or thymus. CCL18, which is constantly present in the serum of healthy subjects, has no counterpart in rodents and its serum level is increased in several benign and malign diseases like lung fibrosis, atopic dermatitis, Gaucher disease, and leukemia [10]–[13]. Some authors also demonstrate that the level of CCL18 is elevated in body fluids and serum of patients with ovarian carcinoma [14], [15]. Examination of tumor tissue located the origin of CCL18 in a subpopulation of TAMs based at the tumor front [16]. Interestingly CCL18 shows some similarity to TGF-ß, but the role of CCL18 in the tumor environment of NSCLC is still unknown. Recently, Chang et al. demonstrated that CCL18 generates regulator T cells, which may help the tumor cells to escape from the immunosurveillance [17]. Therefore we investigated the serum level of lung cancer patients with non-small-cell lung cancer and the correlation of CCL18 to clinical parameters.

## Materials and Methods

### Characteristics of NSCLC Patients Healthy Controls

One hundred and seventy patients diagnosed with NSCLC (UICC Stage I to Stage IV, 6^th^ Edition) and a control group of 31 healthy volunteers are included in this study. Mean age of the patients was 64±10 years, 125 male and 45 female. Adenocarcinoma was diagnosed in 70 patients, 54 patients presented with squamous carcinoma and in 46 cases the examining pathologist described a mixed histology. Staging was performed based on pathological examination where possible; in other cases clinical staging in combination with cytological or histological confirmation of metastasis was performed. Tumor size was estimated using the data given by the pathologists. According to the UICC staging classification (6^th^ Edition) we found 22 patients in stage IA, 19 in stage IB, 5 in stage IIB, 29 in IIIA, 25 in IIIB and 69 in stage IV. In one case the UICC stage was not determined because of insufficient data recordings, which described only the nodal state. The control group consisted of 23 female and 8 male subjects with a mean age of 54±5 years (Tab. 1). Tumor classification according to the histology is shown in Table 2.

**Table 1:** Patients characteristics.

	Patients	Controls
Mean Age	64±10	54±5
Male	125	8
Female	45	23
Smoker	112	
Non-Smoker	44	
Not documented	14	31
Adenocarcinoma	70	
Squamous Carcinoma	54	
Mixed NSCLC	46	
UICC IA	22	
UICC IB	19	
UICC IIB	5	
UICC IIIA	29	
UICC IIIB	25	
UICC IV	69	
UICC not determined	1	

All procedures for informed consent, data collection and privacy protection were approved by the ethic boards of the University Medical Center Freiburg. Clinical data were extracted from the medical record databank of the University Medical Center Freiburg. Serum samples from each individual were obtained at the time of diagnosis during their clinical work-out before any therapeutic treatment was started. Sera were stored at −80°C until analysis was performed. The Diagnosis of NSCLC was histologically or cytologically confirmed. Histological type was determined according to the World Health Organization classification. All tumors were classified according to the UICC 6th Edition Staging procedure included chest X-ray, computed tomography, FDG-Pet/CT, bronchoscopy and/or mediastinoscopy. Pulmonary function tests (PFTs) were routinely performed with a standard methodology at the timepoint before treatment. Overall survival was calculated beginning with the date of diagnosis. Whenever possible, basic information was retrieved by medical records of the University Medical Center Freiburg, from general practitioner or from interviews by telephone. Cross-sectional contact for all surviving patients was performed in April 2011.Mean follow up time was 1325 days.

### Immunodetection of Serum CCL18

Venous blood was sampled using a routine procedure. Blood samples rested 20 minutes before centrifugation. After centrifugation, serum samples were frozen at −80°C and stored. CCL18 was quantified using DuoSet ELISA Development System Kit (R&D Systems Europe, Wiesbaden, Germany). The detection limit for CCL18 ELISA was 7 pg/ml. All samples were measured in duplicate. For duplicate samples, intra-assay coefficients of variation of 10% and inter-assay coefficients of variation of 20% were accepted.

**Table 2:** Tumor classification of the cohort.

	Adeno	SC	Mixed	Total
IA	11	6	5	22
IB	9	8	2	19
IIB	1	4	0	5
IIIA	9	12	8	29
IIIB	8	9	8	25
IV	32	15	22	69
Nd	0		1	1
Total	70	54	46	170

### Statistics

Concentrations are given as median (range) and shown as box plots. Statistical analysis was performed using StatView 5.0 software (SAS Institute, NC). Comparisons between patient groups were performed using ANOVA and Bonferroni-Dunn Test for multiple comparisons. In single comparisons probability values were considered significant if they were less than 0.05. In multiple comparisons levels for p were adjusted for the number of comparisons (Bonferroni). ROC analyses and criterion value plot were performed using MedCalc version 11.6 (MedCalc Software, Belgium). Survival curves were performed according to the method of Kaplan-Meier. Differences in survival were analysed by a log-rank test. Multivariante analysis was performed using the cox proportonal hazards model and correlations were performed using Spearman Rank Correlation.

## Results

### Serum Levels of CCL18 in Patients with NSCLC and Correlation with Clinical and Pathological Parameters

**Figure 1:**
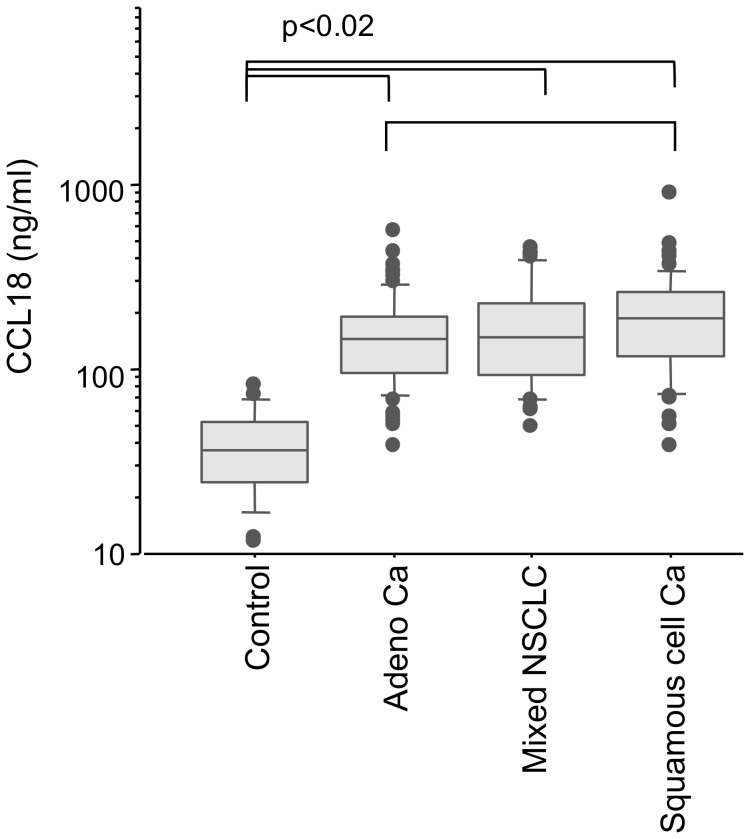
All patient groups disclosed a significantly increased CCL18 serum level compared with controls. There was also a difference between patients with squamous carcinoma and adenocarcinoma (p<0.02). However, this was not significant after Bonferroni correction.

**Figure 2:**
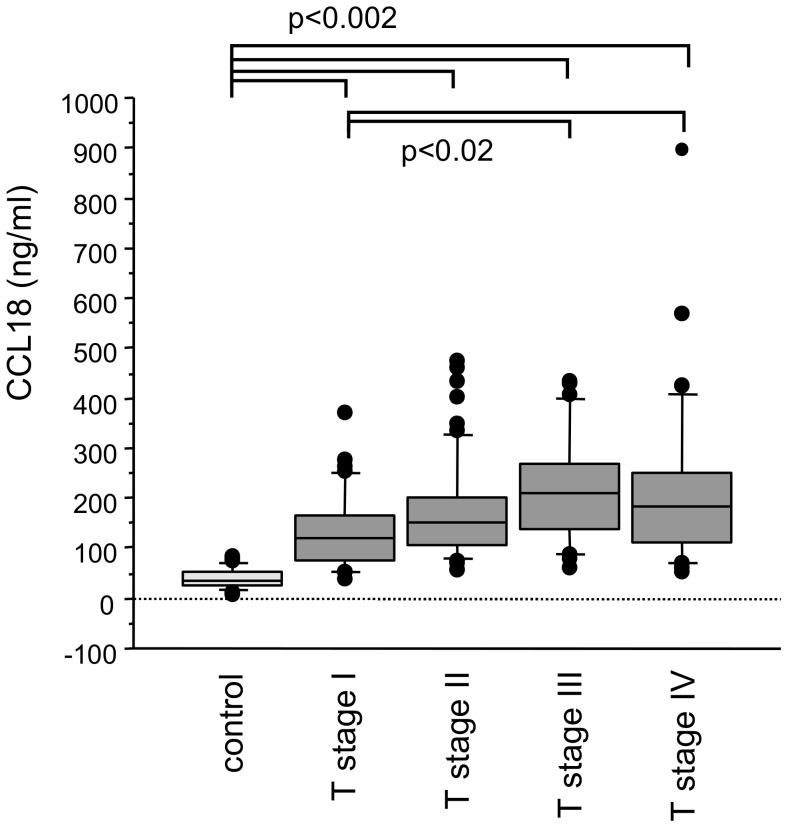
Mean CCL18 levels of all T stages was significantly increased compared with controls (p<0.0002). In addition, there was a significant difference between patient groups with lowest versus the two highest T-stages.

The mean level of CCL18 in the serum samples from carcinoma patients (n = 170) was 150(857) ng/ml, which was significantly higher than the control samples (n = 31, 32(61) ng/ml; p<0.0001). There was a significant difference of all patient groups compared with controls (p<0.0001; Fig. 1). The CCL18 serum level in patients with squamous cell carcinoma was higher than in sera from patients with adenocarcinoma (187(857) ng/ml, 143(528) ng/ml; respectively; p<0.02). CCL18 serum levels increased gradually and significantly with the progress of the T-stage (Fig. 2). Compared with controls CCL18 serum level was significantly higher in T-stage 1 (119(332) ng/ml, n = 39, p = 0.0002) and more so in T-stage 2 patients (150(421) ng/ml, n  = 61 p<0.0001). Comparisons between the tumor patients revealed that levels in T-stage 1 were significantly lower as compared to T-stage 3 (210(374) ng/ml, n = 26, p<0.005) and T-stage 4 (182(845) ng/ml, n = 28, p = 0.002). CCL18 level in T-stage 3 and 4 differed also significantly with controls (p<0.0001). To verify that serum CCL18 concentrations correlate with tumor size we estimated tumor volumes by the pathological data and correlated the tumor volume with serum CCL18 concentrations. Indeed, there was a weak but significant correlation of tumor volume and serum CCL18 (rho = 0.48, p<0.005; Fig. S1). There were no significant differences in the CCL18 serum levels between the different N-stages and M-stages (data not shown).

In univariate analysis there was no correlation between CCL18 serum level and age, neither in the control nor in the patient groups. Adding a second control cohort of significantly younger age (24(18) years) we found a weak but significant correlation of CCL18 with age (rho = 0,45, p<0.0001). However, the difference in CCL18 serum concentration between these two cohorts did not reach a significant level (32(62) ng/ml versus 45(63) ng/ml; not significant; Fig. S2). No significant differences between males and females were found within the subgroups investigated. In addition, multivariate analysis of the data of our tumor patients indicated that CCL18 depends on tumor stage but is independent from age, gender and FEV1 (data not shown).

### Determination of CCL18 Serum Level Cut off Points

**Figure 3:**
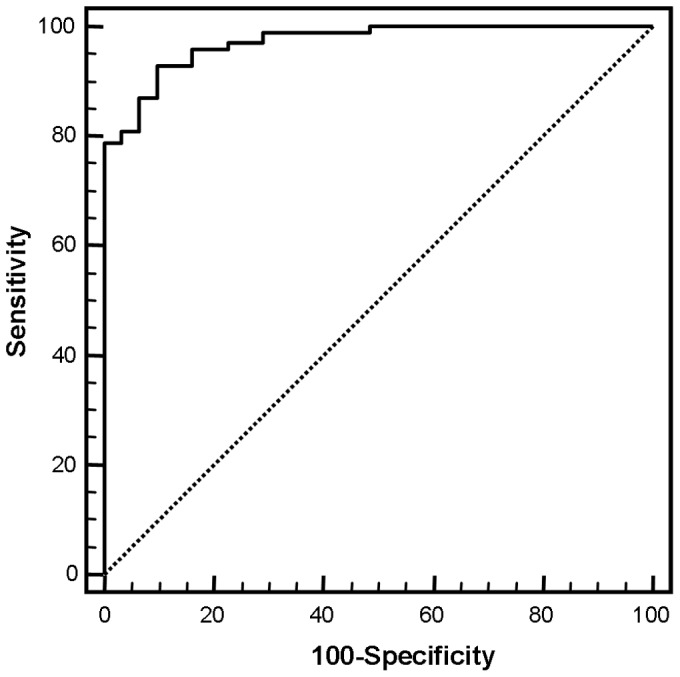
Using a cut-off point of 83 ng/ml ROC analysis revealed an area under curve (AUC) of 0.968 (p<0.0001) indicating a high sensitivity and specificity to discriminate between healthy controls and NSCLC.

Receiver operating characteristic (ROC) analyses revealed a cutoff point of 83 ng/ml (area under the curve (AUC): 0.968; p<0.0001; Fig. 3) to discriminate between healthy controls and NSCLC patients.

**Figure 4:**
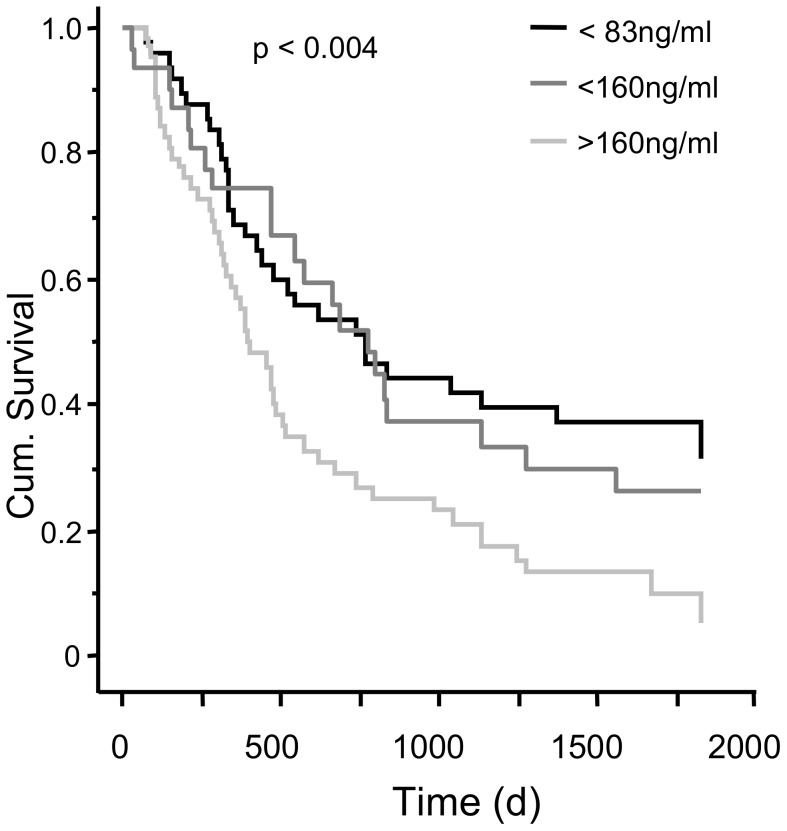
CCL18 serum level and survival of patients with NSCLC in days from time point of diagnosis. Survival time varies significantly with different serum CCL18 levels (p<0.004).

ROC analyses to discriminate between cancer related or non-cancer related death did not lead to a valid AUC. Thus, to further stratify the tumor patients, a criterion value plot was performed leading to a point of equal sensitivity and specificity (54%) of 162 ng/ml. Patients with NSCLC and CCL18 serum level higher than 162 ng/ml had a mean survival time of 623 days, whereas in patients with NSCLC and a serum level between 160 ng/ml and 80 ng/ml mean survival time was 984 days. In the subgroup with a serum CCL18 level of 80 ng/ml or below the mean survival time was 841 days. (p<0.004; Fig. 4).

### CCL18 Serum Level and Survival by Histological Subgroups

**Figure 5:**
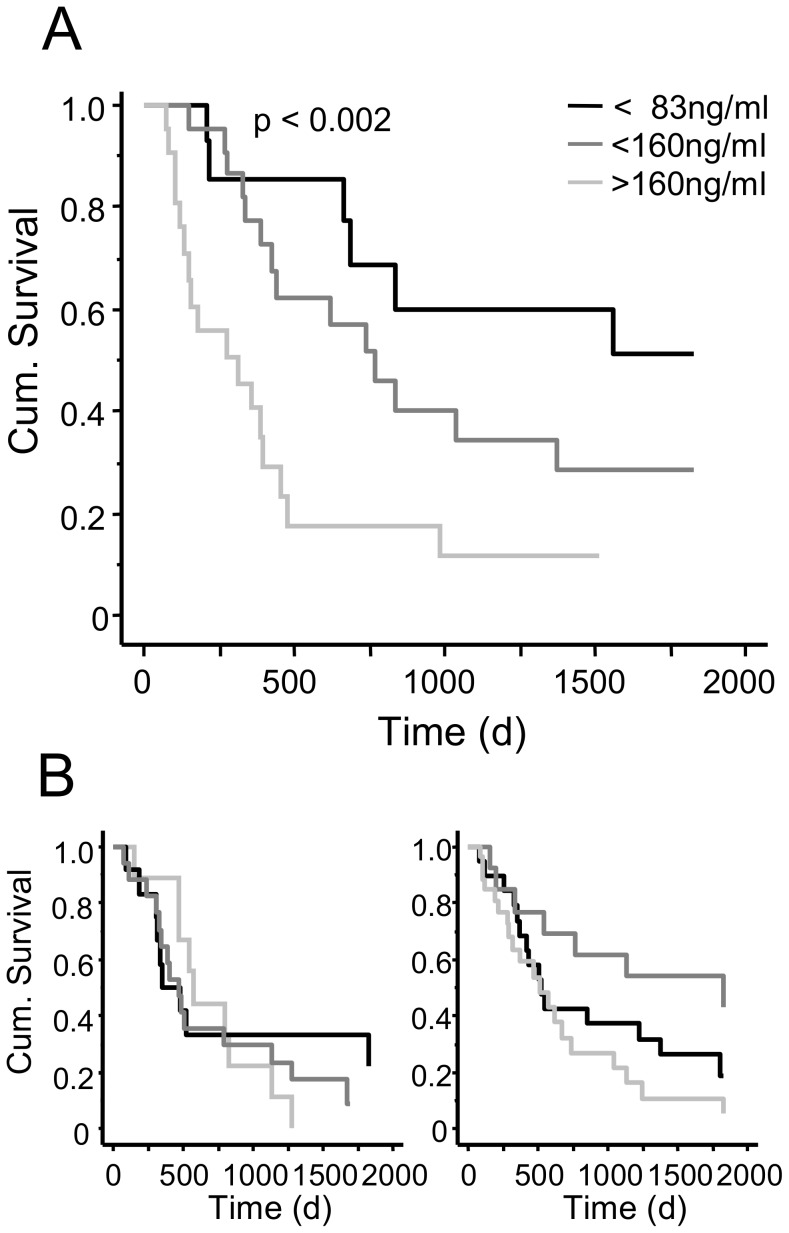
CCL18 serum level and survival time of patients with adenocarcinoma of the lung in days from time point of diagnosis. Survival time of patients with different serum CCL18 levels varies significantly in adenocarcinoma patients (p<0.002; A) but not in patients with squamous carcinoma (B, left panel) and mixed NSCLC (B, right panel).

In patients with adenocarcinoma of the lung we found a mean survival time of 388 days in the group with the highest CCL18 level. In the group with the CCL18 level between 160 ng/ml and 80 ng/ml the mean survival was 788 days and in the group with a CCL18 level below 80 ng/ml the mean survival was 1152 days (p<0.002; Fig. 5A). In contrast we found no significant difference in all three groups if patients suffered from lung carcinoma with mixed histology or from squamous cell carcinoma (Fig. 5B).

### CCL18 Serum Level and FEV1

To exclude the possibility that the observed correlations are caused by COPD which might accompany cancer disease in our patients we also correlated serum CCL18 levels with the forced expiratory volume in 1 second (FEV1) [18]. However, we found no significant differences in FEV1 regarding histology (adenocarcinoma: 84(82)%; NSCLC 77(69)%; squamous carcinoma 76(80)%, not significant, Fig. S3) or T stage (T1∶77(78)%; T2: (79(69)%; T3: (72(60)%; T4∶87(80)%, Fig. S4). In addition, there was no significant correlation between the lung function parameter FEV1 and the CCL18 level in any histological subgroup (adenocarcinoma: rho = −0.02; NSCLC rho = −0.3; squamous carcinoma rho = −0.12, not significant).

## Discussion

Infiltration of tumor associated macrophages (TAM) in lung adenocarcinoma is associated with poor prognosis [19]. In addition, it has been shown that TAM release CCL18 which brought us to the hypothesis that CCL18 might serve as a serum biomarker in patients with lung cancer [16]. We observed that the mean serum level of CCL18 in patients with lung cancer is more than four fold increased compared with healthy controls. Our data demonstrate that CCL18 serum level rises corresponding to the T-stage. In contrast, N- and M-stage did not correlate with CCL18 serum level. Interestingly, the CCL18 serum levels differed according to the histological subtype of lung cancer. Patients suffering from squamous carcinoma disclosed a slightly higher mean CCL18 serum level compared with patients suffering from lung adenocarcinoma, however, this was of minor significance.

CCL18 is a typical product of alternatively activated macrophages and is a possible marker of TAMs [20]. Therefore, we hypothesized that the CCL18 serum level might reflect the number of TAMs. Because TAMs are located within the tumor, their number should increase with the tumor mass. Thus, we analyzed the correlation between CCL18 serum level and T-stage as a surrogate marker for tumor size. This applies in particular in the lower T-stages and indeed the CCL18 serum level increases with increasing T-stages from T-stages 1 to T-stages 3. The main difference between the T-stages 3 and T-stages 4 stage is the infiltration of important mediastinal structures but does not necessarily reflect an increase in size. It was therefore not surprising that no remarkable difference was found in CCL18 serum level of patients with T-stages 3 and T-stages 4. In addition, we found also a weak but significant correlation of serum CCL18 with the tumor volume, indicating that serum CCL18 that tumor size is at least one factor determining CCL18 serum concentrations. However, there are serious constraints in the estimation of tumor volume. Normally, tumors are irregularly shaped and no standard body model (sphere, cylinder or square) reflect real tumor shape. Thus, using the dimensions extracted from the reports by the pathologists to calculate tumor volumes based on a simple, regular body model (length × width × height), the given volumes are only rough estimations of real volumes. This might explain the low correlation between tumor volume and serum CCL18 levels.

N- and M- stages are surrogate markers for hematogenous and lymphogenous metastasis. We could not find a correlation of these parameters with CCL18 serum levels which is also not surprising. The involvement of mediastinal lymph nodes and the formation of metastasis may not or only marginally increase the number of TAMs in a detectable manner.

The fact that we did not find any significant differences regarding the CCL18 serum level in the histological subgroups of patients with non-small-cell lung cancer may depend on the mechanism of CCL18 induction. Despite the different tumor biology of squamous and adenocarcinoma there seems to be a concordant course of induction in both histological subtypes and the potency to foster alternative activation of macrophages and subsequently to induce CCL18 release may be a general property of malignant cells [21]. Currently, the physiological function of CCL18 is thought to affect mainly chemotaxis and homing of leucocytes and the regulation of immunological response [22]. However, recent results of our group and others demonstrate that CCL18 is also involved in the induction of matrix production by fibroblasts [23], [24]. Thus, CCL18 might also be engaged in the formation of the tumor stroma.

TAMs have a strong prognostic value in many malignant tumors [6], [25], [26] as they directly stimulate tumor growth, metastasis, and angiogenesis via release of several growth factors, matrix proteases and cytokines. The tumor-promoting functions of TAMs include also the suppression of adaptive immunity by several mechanisms containing poor antigen presenting capacity and inhibition of T cell proliferation [27], [28]. CCL18 is also capable to generate adaptive regulatory T cells (Tregs) from CD4^+^ CD25^−^ memory T cells [17]. Tregs are physiologically engaged in the maintenance of immunological self-tolerance, but they also inhibit anti-tumor activity of T cells. A large amount of Tregs are present in tumors of patients with different entities and their draining lymph nodes [9], [29], [30]. Importantly the number of Tregs in tumors of patients with lung cancer, breast cancer, gastric cancer and ovarian cancer correlate with poor prognosis and relapse of the disease [31]–[38]. Thus, CCL18 may play a role in the recruitment and the induction of Tregs in the tumor environment and subsequently fosters the escape of the tumor from immune surveillance.

Recently we could demonstrate that CCL18 influences survival of patients in idiopathic pulmonary fibrosis (IPF) [13]. Therefore, we also defined cut-off points and analyzed survival in the NSCLC patients. We stratified the patients using a criterion value plot, which leads us to the CCL18 concentration of 162 ng/ml as a cutoff point. Our ROC analyses revealed a cut-off point with a very high specificity and sensitivity. In contrast, the cut-off point defined by the criterion value plot has only a limited predictive value as demonstrated by the rather low sensitivity and specificity. Nevertheless, using this value as a stratification tool we found striking differences in the mean survival time of NSCLC patients. Patients with a CCL18 concentration of 160 ng/ml or above disclosed a mean survival time which was reduced by one-third compared with patients with a CCL18 concentration less than 160 ng/ml. This effect is most distinctive in the subgroup of patients with adenocarcinoma. Interestingly, Leung et al. estimated CCL18 expression within gastric adenocarcinoma tissue and found an association of high CCL18 mRNA expression in the tumor with prolonged overall survival [16]. In addition, the authors found a positive correlation between CCL18 mRNA expression and T cell markers within the tissue. This is of interest, because CCL18 is described as a T-cell chemoattractant [39]. Thus, increased expression of CCL18 within the tumor fosters the influx of T cells into the tumor and might therefore be protective. In contrast, high levels of CCL18 in the serum might retain T cells within the periphery and inhibits their migration into the tumor.

One might argue that the correlation of good prognosis and lower CCL18 serum concentrations are coincidences rather than causal dependencies because T1 stages which are associated with lower serum CCL18 are R0 resected and consequently do have higher survival rates than other stages. However, although T1 stages in squamous carcinoma or mixed NSCLC are also R0 resected we found a correlation between CCL18 serum level and survival time only in patients with adenocarcinoma but not in squamous carcinoma or mixed NSCLC. Hence, the connection of survival time and serum CCL18 does not depend on different treatment options in these different stages.

Recently, Sin and co-workers demonstrated that CCL18 is increased in chronic obstructive pulmonary disease (COPD) and that this increase is linked with increased total mortality in COPD [18]. Because most of our patients are smokers the presence of COPD might be possible. However, although significant, the increase in CCL18 reported by Sin et al. is much lower as described here. In addition, FEV1 values in our cohorts disclosed a large variability but did not differ significantly between the various histological groups or between the T-stages. Most importantly, CCL18 did not correlate with FEV_1_.

Although our controls are significantly younger than the tumor patients the observed differences between tumor patients and controls are not based solely on age. The observed differences between different age cohorts tested are not significant and the medians of our different control cohorts are located in the data range of controls. Thus, although there might be an influence of age on the CCL18 serum level, the observed differences between controls and tumor patients can not be explained by the different median ages of these groups.

### Conclusions

CCL18 is a marker of alternatively activated macrophages and has therefore an important impact in the orchestra of the “chemokine cross-talk” in the microenvironment of non-small-cell lung cancer. Especially in lung cancer it is of urgent importance to understand how the tumor stroma communicates with the tumor cells and how the chemokines and immune-competent cells influence the process of tumor growth and metastasis. A special aspect of CCL18 is also the immune-regulatory role and the induction of regulatory T cells. This implicates a huge potential of CCL18 to act as an immunosuppressive agent, which plays a key role not only in malignancy but also in benign disease with autoimmune aspects. Strictly limited to the species of primates and lacking in rodents, CCL18 escaped up to now the attention of most investigators.

Our study investigated for the first time the serum level of CCL18 in patients with non-small-cell lung cancer. There is a substantial increase in serum CCL18 levels and ROC analysis demonstrates that CCL18 might be a highly predictive serum marker to differentiate between tumor and non-tumor patients. The correlation between serum CCL18 and tumor stage implies that CCL18 is a surrogate marker for tumour size. CCL18 predict also the survival time in patients with adenocarcinoma of the lung. The presence of highly elevated CCL18 serum levels in patients with non-small-cell lung cancer could point to an important role of CCL18 in the microenvironment of lung cancer and might be a potential target in the therapy of lung cancer.

## Supporting Information

Figure S1 **CCL18 serum concentration (ng/ml) correlates with the tumor volume (cm^3^ as logarithm to the base 10). (rho  =  Spearman rank correlation factor).**
(TIF)

Figure S2 **Median CCL18 levels in two control cohorts of different age.** Controls younger than 45 years (mean age 26±4.5 years) disclose higher CCL18 serum levels than controls with an age of 45 years or higher (mean age 54±4.9 years); however, this difference did not reach statistical significance.
(TIF)

Figure S3 **Forced expiratory volume in one minute (FEV1) in the patient’s cohorts.** All three cohorts disclose a large variation in FEV1, however, there are no significant differences in FEV1 between the cohorts.
(TIF)

Figure S4 **Forced expiratory volume in one minute (FEV1) in the different T stages.** All stages disclose a large variation in FEV1, however, no differences between T stages could be observed.
(TIF)
